# Impact of Ceramic Micropillar Array and Fiber Layer Composite Structure on Kinematic and Heat Transfer Characteristics of Single Droplet Impacting a Wall

**DOI:** 10.3390/mi15040525

**Published:** 2024-04-14

**Authors:** Dechao Zhang, Guangjing Zhang, Yiwei Li, Yaobin Jiang, Yusong Yu

**Affiliations:** Hydrogen Energy and Space Propulsion Laboratory, School of Mechanical, Electronic and Control Engineering, Beijing Jiaotong University, Beijing 100044, China; 21221189@bjtu.edu.cn (D.Z.); 21221190@bjtu.edu.cn (G.Z.); 21221265@bjtu.edu.cn (Y.L.); yaobinjiang131@163.com (Y.J.)

**Keywords:** microcolumn array, droplet impingement, fiber membranes, droplet spreading, Leidenfrost effect, heat transfer

## Abstract

The well-known limitations of spray cooling on high-temperature solids at the Leidenfrost temperature point have been significantly improved by a composite structure of steel micropillar arrays and insulating thin films. However, the physical mechanism of a single droplet impact on the walls of high-temperature composite structures in spray cooling remains elusive. We have experimentally studied and quantified the kinematic and thermal transfer characteristics of a single droplet impacting high-temperature micropillar arrays with fiber membrane composite structures. In particular, micropillar arrays of ceramic materials of different shapes (rectangular and cylindrical) used in this study were made using the more flexible PμSL technique, for which precision reaches the micron level. The results show that the presence and different layouts (embedded or placed on top) of the fiber layer significantly affect the spreading coefficient and thermal transfer efficiency of the droplets after impact. In terms of kinematic characteristics, unrelated to the structure of micropillar arrays, compared to structures without film, the maximum spreading coefficient of droplets significantly increased by more than 40% (43% for rectangular, 46% for cylindrical) when the fiber film was placed on top, and increased by more than 20% (20% for rectangular, 33% for cylindrical) when the fiber film was embedded. In terms of thermal transfer characteristics, at a temperature of 200 °C, the presence of the fiber layer changed the wettability of the surface of the micropillar structure, leading to a certain extension of the total evaporation time of the droplets compared to the surface of the micropillar structure without film.

## 1. Introduction

During the continuous combustion of a high-thrust rocket motor, the gas temperature in the main combustion chamber can reach 3227 °C. High temperatures place high demands on the cooling technology of the walls within the thrust chamber [1,2]. Spray cooling technology has garnered significant interest for its capability to dissipate high levels of heat flux with reduced flow rates, addressing the cooling requirements of thrust chambers efficiently. However, exceeding the critical surface temperature known as the Leidenfrost point, usually below 300 °C on conventional smooth surfaces, leads to the formation of a continuous thin vapor layer beneath the droplet due to rapid vaporization. This insulating layer greatly impairs heat transfer and considerably extends the droplet’s lifetime from less than a second to several seconds. Thus, the Leidenfrost phenomenon notably impacts the effectiveness of spray cooling on highly superheated surfaces.

The parameters of both the droplet and the wall material play crucial roles in influencing the dynamics and heat transfer characteristics of a droplet post-impact. Lipson et al. [3] conducted experiments to explore the impact of pure water and n-heptane droplets on both impermeable and porous stainless-steel surfaces with pore sizes of 5 μm and 100 μm. These experiments revealed that during impact, droplets infiltrate the porous surface, forming a thin film that is subsequently drawn into the pores by capillary action. The rate of droplet diffusion is inversely proportional to the surface roughness. Notably, the Leidenfrost temperature for n-heptane droplets escalates with increased surface porosity, whereas water droplets do not undergo film boiling on porous surfaces. In an effort to delve into the behavior of a single droplet impacting a heated inclined wall, Liu et al. [4] devised an experimental setup to assess the primary reactions of a droplet post-wall impact, varying parameters such as the wall temperature ($T_{w}$ = 40~262 °C), droplet Weber number ($W_{e}$ = 0.66~589), droplet Reynolds number ($R_{e}$ = 189~14,046), and the wall’s inclination angle (α = 0°~45.6°). The findings indicate that elevating the Weber and Reynolds numbers promotes droplet spread rather than contraction. Gravitational effects result in distinct diffusion behaviors of droplets before and after wall impact. Upon contacting a heated surface, if the temperature surpasses a specific threshold, the droplet is suspended atop an evaporating vapor layer. Consequently, the outcome of droplet–wall impact and the thermal exchange between the droplet and surface are significantly governed by the vapor layer’s presence. Recent advancements in measurement techniques have facilitated direct vapor layer assessments, albeit restricted to materials with transparent properties. Eunseop et al. [5] examined the A6061 metal surface, contrasting its boiling heat transfer characteristics against contemporary research findings. Utilizing explicit visual documentation, the study quantitatively and visually analyzed the vapor layer’s recuperation from a transient collapse to immediate re-contact. As a metallic substance, its brief interaction characterized by a substantial heat transfer coefficient predominantly contributes to this observed phenomenon. It has been demonstrated that the heat transfer rate is contingent upon the temperature differential between the droplet’s surface and the saturation point, distinguishing the boiling regime and affirming that the stable vapor layer’s surface temperature is reliant on the impact momentum.

The impact of surface wettability and roughness on the Leidenfrost phenomenon is significant. Chen et al. [6] engineered surfaces with varying wettability and microstructures through physical and chemical etching methods. This study investigated the evaporation behavior and morphological changes of different fuels when they impacted surfaces with varying degrees of roughness. The findings indicated that an increased surface microstructure elevates surface roughness. On lipophilic surfaces, higher surface roughness correlates with a higher Leidenfrost temperature. In contrast, for hydrophilic surfaces, whether inherently or through chemical modification, there is a more pronounced LFP compared to hydrophobic surfaces. This is attributed to the surface’s affinity for liquids, which aids in rewetting under drying conditions. Additionally, Li et al. [7] conducted experimental studies on fluid fragmentation and Leidenfrost dynamics during the impact of droplets on a heated millimeter-scale column. Their results revealed that larger-scale structures increase the Leidenfrost temperature and improve both droplet fragmentation and interfacial heat transfer efficiency during non-isothermal droplet impact.

Recent investigations highlight that surfaces engineered with artificial micro-nano-structures significantly bolster the Leidenfrost phenomenon. This enhancement surpasses improvements attributed solely to changes in wettability from surface roughness [5,8,9,10,11].

The effect of microstructural parameters on the Leidenfrost phenomenon is profound, highlighting the critical role of these dimensions in regulating thermal and physical interactions. Sahoo et al.’s [12] present study revealed substantial Leidenfrost suppression and contact time reduction on a superheated superhydrophobic silicon nanowire (SiNW) array-coated surface. Theoretical modeling revealed that the elevated LFP was caused by a superior capillary force and a strong vapor dispersion into the nanowire array. Datta Prasad et al. [13] reported on the dependence of the Leidenfrost point of a deionized water droplet on the surface morphology. They observe that the LFP increases with the height of micropillars and the spacing between them. Auliano et al. [14,15] demonstrated the preparation of randomly distributed silicon nanowires through metal-assisted chemical etching, with the nanowire height adjustable by varying the etching solution’s exposure times. Findings consistently indicate that the LFP is primarily influenced by the nanostructures’ height. Kwon et al. [16] emphasized the role of surface texturing in elevating the Leidenfrost temperature, focusing on how it enhances droplet wetting through capillary water absorption under conditions of high superheat. Intriguingly, the study reveals that sparser textures, as opposed to denser ones, are more effective in increasing the Leidenfrost temperature. Kim et al. [17] presented a comprehensive review of recent literature concerning the Leidenfrost drops on micro/nanostructured surfaces with an emphasis on the enhancement of the Leidenfrost temperature. It was concluded that multiscale hierarchical surfaces hold the best promise to significantly boost the Leidenfrost silicon-based structured surfaces, commonly employed in investigating the LFP, which have been extensively studied. In summary, the design of the microstructure size and arrangement is crucial in postponing the onset of the Leidenfrost point.

Jiang et al. [18] introduced a structured thermal armor featuring an array of steel microcolumns and integrated thermal insulation membranes. This design effectively suppresses the Leidenfrost phenomenon, achieving the highest reported LFP threshold of up to 1150 °C in the literature. The position of the fiber layer was shown to have a significant effect on the increase in LFP points. The arrangement of the fiber layer, whether embedded or surface-mounted, markedly influences the enhancement of the LFP. However, previous studies have not quantitatively analyzed the impact of microcolumn arrays and composite structures with varying fiber layer positions on the kinematic properties (e.g., spreading coefficients) of single droplet impingement. Additionally, the heat transfer characteristics of a single droplet impacting composite structures have not been thoroughly examined. It is noteworthy that the discussed micropillar arrays, fabricated using milling techniques, result in material waste and limit both the materials and shapes available for the micropillar arrays.

In this study, two types of micropillar arrays of ceramic materials at the 100 μm level, rectangular and circular, were obtained using the PμSL technique. A visualization test platform of the droplet-impacted micropillar arrays and superposition of fiber layers was conducted. The effects of different micro-pillar array shapes and fiber layer arrangements on the kinematic and heat transfer characteristics of a single droplet impinging on the wall were quantitatively studied and analyzed with a high-speed camera. This is of guiding significance for clarifying the droplet kinematics and wall heat transfer characteristics of droplets hitting high-temperature walls in spray cooling and for improving the efficiency of the spray cooling design.

## 2. Materials and Methods

Droplet impact is a transient process, and determining how to obtain the changes in the kinematic properties of droplets during this transient process, such as spreading, splashing, retraction, and fragmentation, is very important for understanding the droplet impact process and conducting experiments. In this chapter, we will first set up the experimental platform for droplet impact experiments according to the experimental scheme and conditions, capture the process of droplet impact with high-speed photography, introduce the preparation methods of micrometer fiber membranes and microcolumn arrays, show the advantages of the comparative analysis of the fabrication methods of the microcolumn arrays, and finally introduce the experimental errors and uncertainties.

### 2.1. Droplet Impact Test Platform and Experimental Equipment

**Figure 1:**
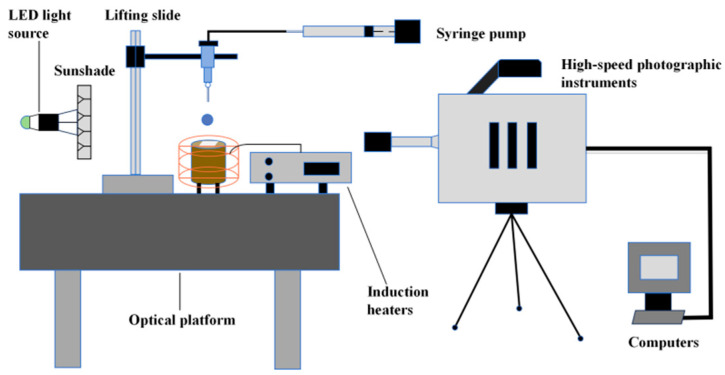
Schematic diagram of droplet impact test system.

Figure 1 shows the schematic diagram of the experimental platform for the droplet impact wall. The platform consists of a hardware system composed of a generation device, an acquisition system, a surface heating system, and a bench support system. The software system is composed of image acquisition, droplet generation control, and temperature acquisition systems. During the experiment, a high-speed camera, model PHOTRON Nova S9 by Photron Limited, Tokyo, Japan, with a maximum resolution of 1024 × 1024, was arranged on the front of the impact surface. A macro lens, Nikon 85 mm f/2.8 1-5X Super Macro by Shenyang ZHONGYI Optical Technology Co., Ltd., Shenyang, China, was mounted on the high-speed camera, and the high-speed camera was connected to the computer through a network cable interface, so that the pictures obtained from the shooting could be transmitted and stored in real time.

**Figure 2:**
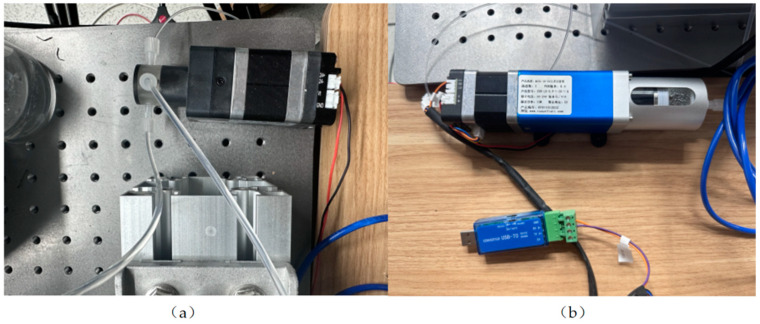
(**a**) High-pressure 3-way valve (Mrv-01B-T03-K3.0-S-M05); (**b**) Syringe pump (ZSB-LS-0.9-1-20-1-Q).

Figure 2 shows the hardware system devices mainly used in the experiment. The inlet of the high-pressure three-way valve is connected to the micro syringe pump, and the outlet is connected to the container where the work material is stored. By changing the state of the three-way valve, the micro syringe pump can be made to extract or discharge the liquid. When liquid is present in the micro syringe pump, the motor connected to it slowly pushes the liquid through the three-way valve and then to the needle to form a droplet. In addition, different droplet impact velocities can be obtained by fixing the needle at different heights.

**Figure 3:**
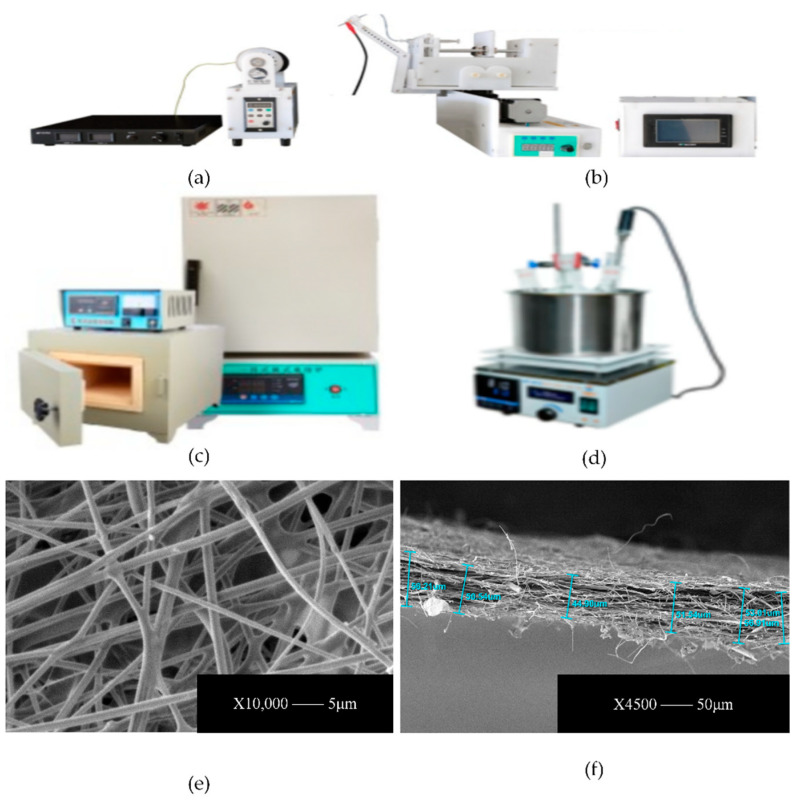
(**a**,**b**) The electrostatic spinning main experimental equipment; (**c**) The XH3L-17-type ceramic fiber muffle furnace; (**d**) The DF-101A collector-type magnetic stirrer; (**e**,**f**) The SiO_2_ nanofiber film after calcination.

Figure 3 shows a device diagram of electrostatic spinning process. The polyvinyl alcohol powder was dissolved in deionized water at 25 °C, and the beaker solution was placed in a magnetic stirrer for waterside heating and stirring in 80 °C warm waters and stirred continuously at a constant temperature for 12 h to produce PVA precursor solution. Then, phosphoric acid was used as the hydrolysis catalyst with tetraethoxysilane and water in accordance with a certain molar ratio at room temperature. Continuous stirring was performed for 10 h to obtain silane sol. Finally, the prepared silane sol and the PVA precursor solution with the same mass were mixed and then continuously stirred at room temperature for 4 h to obtain a homogeneous precursor solution. The solution was put into a 10 mL syringe and injected at a flow rate of 0.5 mL h^−1^ via a micro syringe pump. Under the influence of electrostatic forces from a high-voltage power supply, the collector underwent electrospinning. Subsequently, a micron-scale silica fiber membrane was produced through air calcination in a muffle furnace. The surface, internal structure and thickness of this silica material were scrutinized using a SEM3200 scanning electron microscope manufactured by Guoyi Quantum Technology (Hefei) Co., Ltd., Hefei, China. The thickness of the fiber membrane was determined at various measurement points, from which an average thickness of 62.51 μm was calculated. This measurement was established as the standard thickness for the fiber membrane. Moreover, it was observed that the thickness of the fiber layer could be effectively controlled by adjusting the duration of the spinning process.

During the fabrication of micropillar arrays, it was considered that the chambers could be insulated using high-temperature-resistant ceramics. For this purpose, UV-cured 3D-printing technology, which is known for its ability to achieve processing accuracy of 10 μm to 25 μm, was employed in this study. Subsequently, the ceramic paste was cured by UV irradiation and then sintered at elevated temperatures to achieve the desired structural integrity and properties.

Importantly, 3D printing technology offers significant advantages over methods such as milling and chemical etching, which are also capable of producing micropillar arrays. Milling, which typically involves complex machining procedures using a milling machine, is not only labor intensive but also results in material waste due to the cutting process and environmental pollution if waste disposal measures are not taken properly. In contrast, 3D-printing technology allows for rapid prototyping and production of parts, greatly reducing material consumption and minimizing waste. In terms of chemical etching, a study by Bibiana Vogel Peres Riesgo et al. [19] has shown that too high an acid concentration or too long an etching time significantly reduces the strength of silicate ceramic compositions. In addition, comprehensive studies on the effect of etching protocols on the mechanical properties of ceramics are still insufficient, introducing considerable uncertainty. In addition, chemical etching processes require safety considerations, which can lead to incomplete or uneven etching, further complicating the process. In contrast, 3D-printing methods offer significant material and environmental advantages. They mitigate the safety concerns associated with chemical processes and circumvent the additional costs typically associated with ensuring safety measures. In addition, 3D-printing technologies facilitate environmentally sustainable manufacturing practices, consistent with contemporary efforts to minimize environmental pollution.

**Figure 4:**
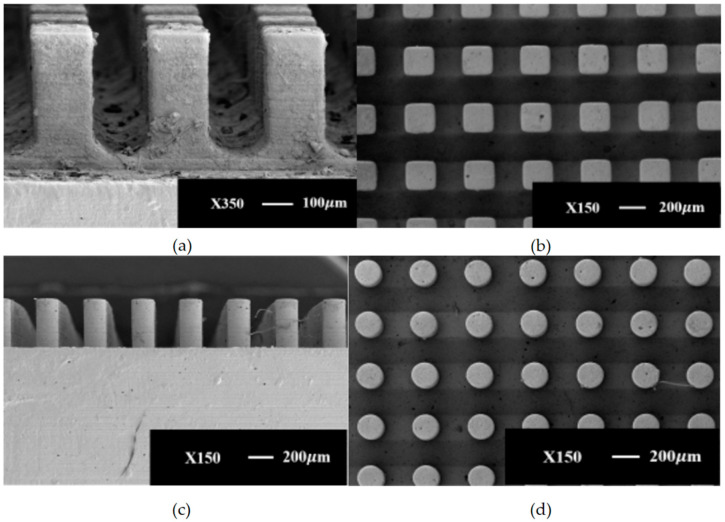
Scanning electron microscope images (SEM 3200) of different ceramic micropillar array structures using the Pμsl technique: (**a**,**b**) 200 μm × 200 μm × 450 μm rectangular microcolumn arrays; (**c**,**d**) 450 μm × 200 μm × 400 μm cylindrical microcolumn arrays.

Figure 4 shows the SEM images of different micropillar array structures. It can be observed by SEM that the walls of different micropillar array structures show regular arrangement.

### 2.2. Measurement Methods

Droplet impact wall experiments use high-speed photography to measure the evolution of the dynamic behavior of the process. A K-type thermocouple wire was used to determine the wall temperature, and ceramic internal temperature measurements were made to determine the absolute temperature of the two measurement points using the formula for calculating the heat flow density.

**Figure 5:**
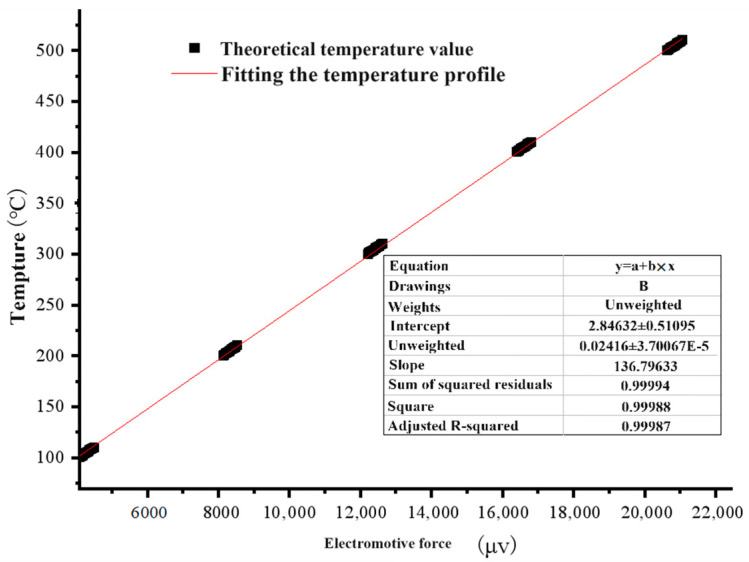
Fitted curve for thermocouple temperature calibration.

Figure 5 shows the fitted curve for the temperature calibration of the thermocouple. The thermocouple’s temperature curve is fitted as a function of

y = 0.0242x + 2.8463.(1)

### 2.3. Experimental Error and Uncertainty Analysis

The inaccuracies observed in droplet impact experiments predominantly stem from errors in thermocouple temperature measurements and dimensional discrepancies. The uncertainties are primarily related to the physical properties of the droplet and parameters associated with heat transfer [20,21,22,23].

(1) Error analysis

In single-droplet wall experiments, the error sources primarily include three main aspects: measurement device error, environmental error, and measurement method error. The error from the measurement device is largely produced by the thermocouple. An examination of the thermocouple’s tolerance table reveals that the temperature measurement error range for the thermocouple used in the experiment is ±1.5 °C. The size error of the thermocouple mainly arises from the dimensional inaccuracies of its temperature measurement hole. By using vernier calipers to gauge the experimental samples and the temperature measurement holes, a manufacturing precision of ±0.1 mm is achieved. The environmental error mainly pertains to the ongoing fluctuations in temperature, air pressure, and air humidity within the observation environment, contributing to inaccuracies in the measurement results. In this experiment, given its short duration, fixed location, and simultaneous measurement and documentation of these variables during the experiment, the influence of environmental errors on the results is considerably minimized. Regarding the measurement method, the experiment utilizes a stepper motor module to control the initial distance between the wall and the droplet and a micro-pump to regulate the droplet’s delivery speed and frequency, thus ensuring the droplet’s size and velocity upon contact with the wall are kept within the permissible error range.

(2) Uncertainty analysis

Building upon the analysis of error sources outlined above, this study employs the Holman [24] methodology to assess the experimental system’s uncertainty. For parameters measured directly, their measurement errors can be straightforwardly derived from the precision of the measuring instruments. However, for indirectly measured parameters—those deduced through calculations based on parameters measured directly—error must be determined through error propagation.

For directly measured parameters, the uncertainty is expressed as

(2) $$x_{i}=x_{i,meas}\pm \delta_{x}$$

If the parameter *y* is satisfied $\begin{array}{c} y=f\left(x_{1},x_{2},\ldots \ldots ,x_{n}\right), \end{array}\;$ and if the parameters affecting *y* are all independent of each other, then the absolute uncertainty in *y* can be obtained from Equation (3):

(3) $$\sigma_{y}=\sqrt{\sum_{i=1}^{n}\;{\left(\frac{\partial f}{\partial x_{i}}\cdot \sigma_{x_{i}}\right)}^{2}}$$

We can determine the relative uncertainty for each parameter based on the measurement instruments, methodologies, and data analysis techniques utilized in the experiments.

Relative uncertainty of the initial distance of the droplet from the wall: the distance from the single droplet generation probe to the wall is ∆l = 255 mm, $\sigma_{∆l}$ = 1 mm. Thus, the relative uncertainties are, respectively:

(4) $$\frac{\sigma_{∆l}}{∆l}=0.39\%$$

Relative uncertainty of droplet wall impact time: the total duration for the droplet to travel from the probe to the wall is recorded as T = 0.228 s, and the moment of the droplet’s impact on the wall within the experiment is identified by the final image captured before impact. With the high-speed camera (PHOTRON Nova S9) configured to a frame rate of 4000 fps, the time gap between successive frames is calculated as $\sigma_{t}$ = 1/4000 s = 0.25 ms. Then, the relative uncertainty can be expressed as follows:

(5) $$\frac{\sigma_{∆t}}{∆t}=0.11\%$$

Relative uncertainty of wall temperature: the temperature measurement error associated with the thermocouple utilized in the experiment is known to be within a range of ±1.5 °C ($\sigma_{tw}$), with the minimum measured wall temperature ($T_{w}$) recorded at 200 °C. Consequently, the relative uncertainty is determined as follows:

(6) $$\frac{\sigma_{tw}}{T_{w}}=0.75\%$$

The velocity of the droplet at impact is ascertained using the final two frames captured before the impact occurs. With the high-speed camera’s sampling rate adjusted to 4000 fps, the temporal gap between these two frames is ∆t = 1/4000 s = 0.25 ms. The impact velocity, denoted as v, is then computed by calculating the displacement of the droplet’s center, ∆l, in these last two frames before the droplet makes contact with the wall. Relative uncertainty of droplet velocity can be expressed as follows:

(7) $$v=\frac{∆l}{∆t}$$

(8) $$\sigma_{v}=\sqrt{{\left(\frac{1}{∆t}\sigma_{∆l}\right)}^{2}+{\left(\frac{∆l}{{\left(∆t\right)}^{2}}\sigma_{∆t}\right)}^{2}}$$

(9) $$\frac{\sigma_{v}}{v}=\sqrt{{\left(\frac{1}{v}\frac{1}{∆t}\sigma_{∆l}\right)}^{2}+{\left(\frac{1}{v}\frac{∆l}{{\left(∆t\right)}^{2}}\sigma_{∆t}\right)}^{2}}=0.20\%$$

where ∆t=0.2281 s, ∆l=0.255 m, $\sigma_{∆l}=1\;mm$, and $\sigma_{∆t}=2.5\times 10^{-4}\;s$.

Given that the droplet does not maintain a perfectly spherical shape throughout its descent, the diameter of the droplet is represented by its equivalent diameter, $D_{e}$, where $D_{h}$ and $D_{v}$ are the horizontal and vertical diameters of the droplet, respectively [4]. In this experiment, pixel analysis was employed to measure the geometric characteristics of the droplets. For droplets of consistent size, camera settings (such as focal length and aperture opening) were standardized to photograph a calibrator of known dimensions. This process established a relationship between pixels and real-world measurements, allowing for the determination of actual lengths by assessing the pixel count in the images. $\sigma_{D_{h}=}\sigma_{D_{v}}$ is the length of a single pixel point size. Then, the relative uncertainty of droplet size can be expressed as follows:

(10) $$D_{e}=\sqrt[{3}]{{D_{h}}^{2}\cdot D_{v}}$$

(11) $$\sigma_{D_{e}}=\sqrt[{2}]{{\left[\left(\frac{3}{2}{D_{h}}^{\frac{1}{2}}\cdot {D_{v}}^{\frac{1}{3}}\right)\cdot \sigma_{D_{h}}\right]}^{2}+{\left[\left(\frac{3}{2}{D_{h}}^{\frac{3}{2}}\cdot {D_{v}}^{-\frac{2}{3}}\right)\cdot \sigma_{D_{v}}\right]}^{2}}$$

(12) $$\frac{\sigma_{D_{e}}}{D_{e}}=\sqrt[{2}]{{\left[\left(\frac{1}{D_{e}}\frac{3}{2}{D_{h}}^{\frac{1}{2}}\cdot {D_{v}}^{\frac{1}{3}}\right)\cdot \sigma_{D_{h}}\right]}^{2}+{\left[\frac{1}{D_{e}}\left(\frac{3}{2}{D_{h}}^{\frac{3}{2}}\cdot {D_{v}}^{-\frac{2}{3}}\right)\cdot \sigma_{D_{v}}\right]}^{2}}=0.03\%$$

where $\sigma_{D_{h}}=\sigma_{D_{v}}=2.48\times 10^{-5}\;m$, $D_{h}=2.1\;mm$, and $D_{v}=1.8\;mm$.

Relative uncertainty of heat transfer coefficient: in the experiment, we utilized a high-frequency induction heater, model HF-3000, characterized by an instrument control power accuracy of ±5%. The experimental heating power, denoted as P, was measured at 12.73 KW, while the circular area, with a diameter of $D_{e}$, served as the area for ideal heat transfer (the maximum spreading factor of the droplets in the experiment is about 4).

(13) $$q=\frac{P}{A}$$

(14) $$A=\frac{\pi {{(4D}_{e})}^{2}}{4}$$

(15) $$q=\frac{1}{4\pi }\cdot \frac{P}{{D_{e}}^{2}}$$

(16) $$\frac{\sigma_{q}}{q}=\sqrt{{\left(\frac{1}{q}\frac{1}{4\pi }\cdot \frac{1}{{D_{e}}^{2}}\cdot \sigma_{P}\right)}^{2}+{\left(\frac{1}{q}\frac{1}{4\pi }\cdot \frac{2P}{{D_{e}}^{3}}\cdot \sigma_{D_{e}}\right)}^{2}}=2.34\%$$

where $D_{e}=1.99\times 10^{-3}\;m$, $\sigma_{P}=2.5\%\times 12.73=318\;W$, $\sigma_{D_{e}}=6.65\times 10^{-7}\;m$, and P=12.73 KW.

Upon conducting the aforementioned calculations, we can subsequently compile Table 1, which encompasses error and uncertainty analysis.

## 3. Results and Analysis

This chapter delves into the visualization of droplet motion behavior, specifically examining the impact of a single droplet on a microcolumn array and a layered fiber structure. It characterizes the behavior of the droplet post-impact by measuring the spread diameter of the droplet and then converting it into a dimensionless spreading coefficient for analysis. This study also measures the internal temperature of the ceramic sheet and the temperature of the wall to calculate the heat flux density values for different wall structures. This allows for a comparative analysis of the wall’s heat transfer characteristics following droplet impact under various experimental conditions.

### 3.1. Influence of Fiber Layer Arrangement on Droplet Kinematic Properties after Single-Droplet Impacts

This section provides an in-depth examination of the wall structures under study, focusing on micropillar array configurations, including rectangular and cylindrical micropillar arrays, with specific structural parameters outlined in Table 2. For ease of reference, the rectangular micropillar array is designated as Sample 1, while the cylindrical micropillar array is referred to as Sample 2. Specifically, the rectangular micropillar array features micropillars with a height and width of 200 μm and a spacing between micropillars of 450 μm. Conversely, the cylindrical micropillar array is characterized by micropillars with a height of 450 μm, a diameter of 200 μm, and a spacing of 400 μm. Table 2 details the structural parameters of the various micropillar arrays implemented on the wall.

Table 3 shows the test conditions and their related parameters.

Droplets, each with a diameter of 2 mm, were released from a height of 255 mm above the wall, targeting various microcolumn array structures (rectangular and cylindrical arrays) at a wall temperature of 200 °C and achieving an impact velocity of 2.23 m/s. The initial frame captured immediately before the droplet’s contact with the wall serves as the reference point for analyzing the droplet’s kinematic behavior throughout the impact process.

To enhance the analysis of the droplet’s spreading behavior upon impact, this study employs dimensionless parameters, enabling a quantitative evaluation of the droplet’s transient dynamics as it interacts with the wall. These dimensionless parameters include the spreading diameter coefficient D*, height coefficient H*, and time t*, each defined as follows:

(17) $$D^{*}=\frac{D_{t}}{D_{0}}$$

(18) $$t^{*}=\frac{t_{0}v}{D_{0}}$$

where $D_{t}$ represent the instantaneous spreading diameter upon impact on the surface, respectively. The variable $t_{0}$ signifies the time elapsed during droplet spreading. Furthermore, v indicates the droplet’s impact velocity, and $D_{0}$ corresponds to the droplet’s initial diameter.

**Figure 6:**
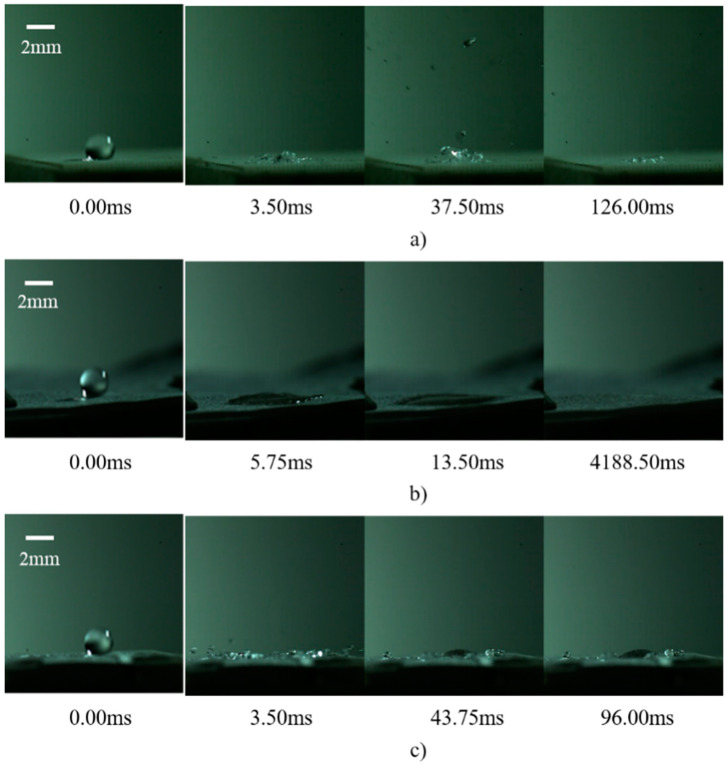
Droplet images after a single droplet impacts a cylindrical array of fiber layers in different arrangements: (**a**) no fiber membrane; (**b**) fiber membrane placed on top; (**c**) fiber membrane placed inside.

Figure 6 presents the sequence of images capturing the behavior of a single droplet upon its impact on a rectangular micropillar array, modified by different configurations of fiber layers. When the wall temperature is maintained at 200 °C, the impact on the rectangular micropillar array induces a series of dynamic responses, including droplet splashing, the formation of a central jet, bubble dynamics (expansion and collapse), and the oscillation of the liquid film. These phenomena can be attributed to the transient modulation of interaction forces between the droplet and the micropillar surface, which, in turn, initiates pressure differentials across the droplet surface. Such differentials are responsible for the observed complex behaviors, including jetting and bubbling on the droplet surface.

Upon the integration of a silica micron fiber film atop the micropillar structure, if the fiber layer solely overlays the micropillar surface, it acts as a dampener, mitigating the formation of high- and low-pressure zones on the droplet surface. This suppression effect curtails the droplet splashing and jetting phenomena significantly, predominantly leading to the spread and subsequent evaporative drying of the liquid film instead.

Conversely, embedding the fiber film within the micropillar framework using an inverse mold technique alters the surface texture of the micropillars, increases the droplet-surface contact area, and enhances droplet adhesion. These modifications reduce the splash height upon impact, increase the size of splash droplets, and promote droplet adhesion. Additionally, the incorporation of the fiber layer impedes droplet mobility across the micropillar surface, thereby extending the droplet’s dwell time.

**Figure 7:**
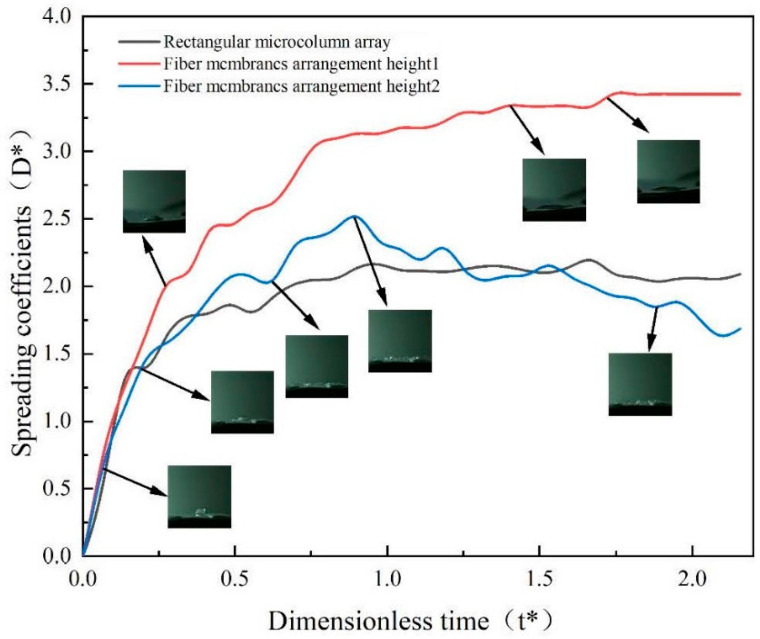
Variation in droplet spreading coefficients for different arrangements of rectangular microcolumn array fiber membranes.

Figure 7 illustrates the changes in the droplet spreading coefficient across various configurations of the rectangular micropillar array with integrated fiber membranes. The analysis reveals a variation in the droplet spreading coefficient with the strategic placement of the fiber membrane. Specifically, positioning the fiber membrane atop the micropillar structure elevates the spreading coefficient to 3.5, marking a 43% increase over the baseline spreading coefficient of 2.0 observed in the rectangular micropillar array without a fiber membrane. This significant enhancement can be attributed to the direct impact of the droplet on the hydrophilic surface of the fiber membrane.

Further intricacies emerge when the fiber membrane is embedded within the micropillar structure. Under these conditions, the spreading coefficient of the droplet impacting the rectangular micropillar array exhibits a correlation with the dimensionless time post-impact, achieving a peak value of 2.5. This represents a 20% improvement compared to impacts on structures lacking a membrane, yet it shows a 23% reduction when compared to the coefficient obtained when the membrane is placed directly atop the structure. These findings underscore the critical role of the fiber membrane in augmenting the spreading coefficient. However, for optimizing both droplet impact rebound and thermal efficiency of the wall surface, positioning the fiber membrane at a certain height proves to be more advantageous. While the placement of the fiber membrane on the micropillar surface undeniably increases the droplet contact area, it potentially hinders steam flow beneath and suppresses dynamic behaviors such as droplet splashing, primarily due to the droplet being absorbed by the hydrophilic fiber membrane.

**Figure 8:**
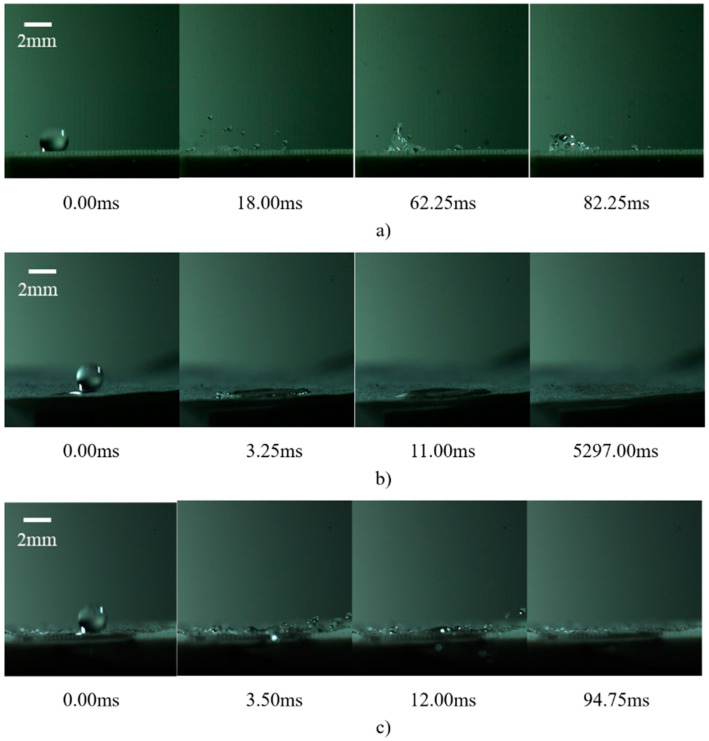
Droplet images after a single droplet impacting a rectangular array of fiber layers in different arrangements: (**a**) no fiber membrane; (**b**) fiber membrane placed on top; (**c**) fiber membrane placed inside.

Figure 8 depicts the sequence of droplet behaviors following impact on cylindrical micropillar arrays under various arrangements, with the impacts occurring at a velocity of 2.23 m/s and a wall temperature of 200 °C. The initial interaction of the droplet with the cylindrical micropillar array’s surface does not significantly alter its trajectory or integrity. However, as a consequence of the micropillar surface geometry, fragmentation and splashing of the droplet are observed post-impact. Over time, the interaction dynamics evolve; droplets at the base of the structure are levitated by vapor generated at the interface, while those at the apex tend to detach due to reduced surface tension.

The observation period further reveals the formation and subsequent fragmentation of large bubbles at the base, ascending vertically before disintegrating into smaller droplet splashes. This process facilitates the gradual vaporization of the droplets. It is noted that certain droplets remain suspended above the micropillar array, influenced by the steam generated beneath them. Upon impact with the fiber membrane atop the structure, the inherent hydrophilicity of the membrane leads to the spreading of droplets until complete evaporation. Embedding the fiber membrane within the micropillar structure introduces a combined effect of structural adhesion and edge wetting, whereby peripheral droplets are absorbed by the synergistic interaction between the micropillar array and the overlying fiber layer, promoting adhesion to the structural wall.

**Figure 9:**
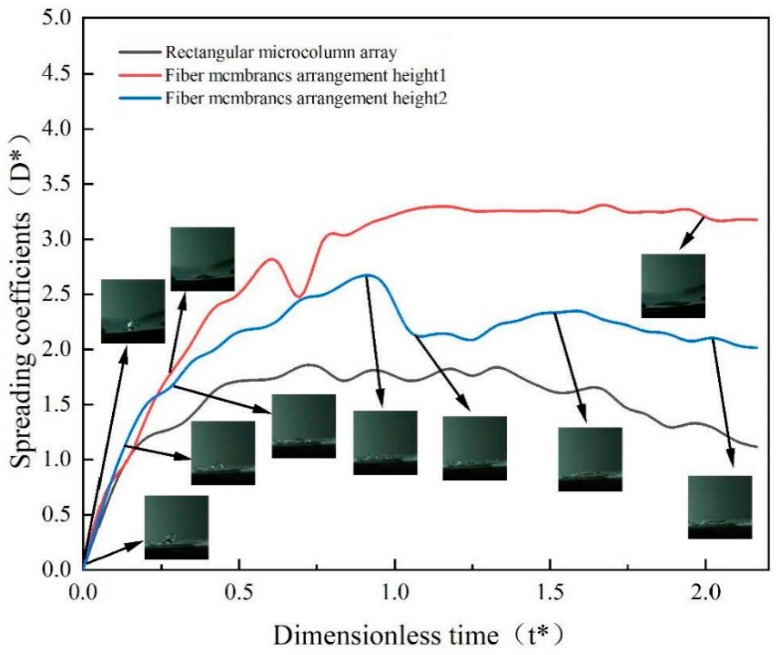
Variation in droplet spreading coefficient for different arrangements of cylindrical microcolumn array fiber membrane.

Figure 9 illustrates the variations in the spreading coefficient of droplets impacting cylindrical micropillar arrays, differentiated by the arrangement of fiber membranes. For the array without any fiber membrane, the spreading coefficient initially ascends to a peak value of 1.75, maintains stability for a brief interval, and then exhibits a gradual decline as dimensionless time progresses. In contrast, when the droplet impacts a fiber membrane positioned atop the columnar wall, the spreading coefficient progressively increases with dimensionless time, ultimately stabilizing at 3.25. Furthermore, the impact on a fiber membrane embedded within the micropillar array elevates the peak spreading coefficient to 2.6, marking a 33% increase over the maximum value observed in the absence of a membrane. Notably, the presence of a fiber membrane atop the microcolumn structure boosts the spreading coefficient to 3.6, representing a 46% increment compared to the non-membrane setup, albeit a 13% decrease relative to the configuration at a lower arrangement height.

The kinematic behavior of droplets exhibits a pronounced distinction at different arrangement heights. At a higher arrangement height, small droplets demonstrate adherence to the surface of the microcolumn structure, indicative of a more complex interplay of forces. Conversely, at the lower height, the droplet dynamics are predominantly characterized by spreading upon impact with the fiber membrane atop the cylindrical array. This behavior can be attributed to the hydrophilic nature of the fiber membrane, which significantly influences the droplet’s contact angle.

### 3.2. Influence of Fiber Layer Arrangement on Wall Heat Transfer Characteristics after Single-Droplet Impingement

The test was conducted at a temperature of 200 °C with a droplet of 2 mm in diameter falling from the tip of the needle at a distance of 255 mm from the wall. Samples 1, 2, 3, and 4 were made of high-temperature ceramic material with a base size of 24 mm × 24 mm.

Table 4 shows the physical parameters of high-temperature ceramics. In this experiment, high-temperature ceramic material was used as the base, and aluminum oxide was used as the ceramic slurry for 3D printing. Aluminum oxide was chosen as the ceramic slurry for 3D printing. The density of the 3D-printed ceramic was 3819.40 kg/m^3^. Its specific heat capacity was 2.27 KJ/(kg·K), and its thermal conductivity was 1.79 (W/m·K).

Upon impact of the droplet on a smooth plane, the temperature distribution within the ceramic sheet tends to stabilize within 0.4 s, resulting in a relatively smooth temperature curve inside the ceramic sheet. Moreover, the instantaneous change in wall temperature is virtually negligible. The heat flux density can be calculated using the following formula:

(19) $$q=\frac{P}{A}=\frac{\lambda ∆T}{L}$$

where ∆T denotes the temperature difference between two measurement points on the thermocouple, and λ represents the thermal conductivity of the high-temperature ceramic samples, measured at 1.4 W/(m·K). *L* is the vertical distance from the temperature measurement point inside the ceramic to the bottom of the ceramic slice.

**Figure 10:**
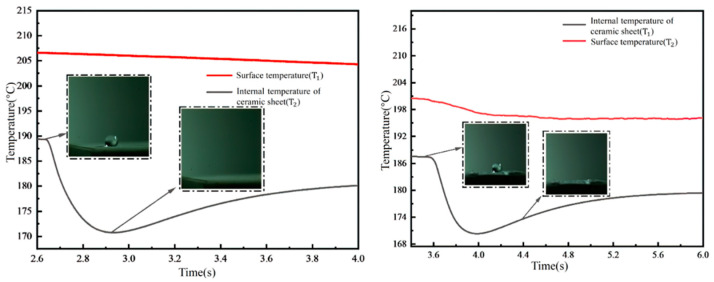
Temperature variation of droplet impacting rectangular micropillar arrays with fibrous layer superimposed structures at 200 °C.

Figure 10 delineates the temperature variations observed when a single droplet impacts a rectangular micropillar array overlaid with a fiber layer. Upon impact, part of the droplet permeates through to the bottom of the ceramic sheet, facilitated by the presence of the fiber membrane. Due to the cooler temperature of the droplet, heat transfer occurs from the micropillar structure to the droplet. Consequently, a distinct step change in the internal temperature of the ceramic sheet is observed, continuing until the droplet completely evaporates, after which the internal temperature rises and eventually stabilizes. This behavior can be attributed to the low thermal conductivity of the ceramic sheet and the hydrophilic nature of the fiber membrane, which absorbs the droplet upon impact. Furthermore, when the temperature reaches the Leidenfrost point, the inability to form a vapor film leads to a slight step-like change in the wall temperature as well. Quantitatively, in the presence of the fiber membrane structure, the internal temperature of the ceramic sheet exhibits a reduction from 187 °C to 171 °C within approximately 0.4 s. Simultaneously, the wall temperature decreases marginally from 200 °C to 197 °C. Employing the heat flow density formula, the heat flux density under these conditions is calculated to be approximately 13,375 W/m^2^.

**Figure 11:**
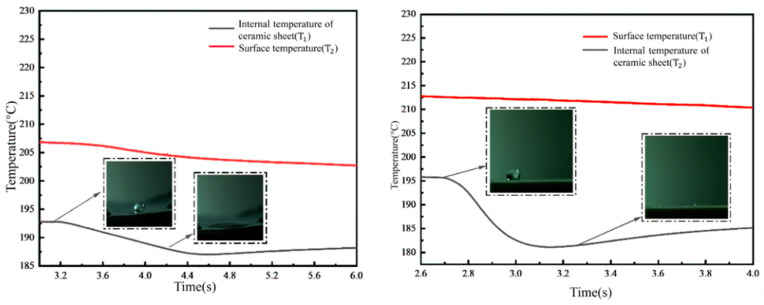
Temperature variation of droplet impacting cylindrical micropillar array with fibrous layer superimposed structure at 200 °C (membrane unmounted).

Figure 11 illustrates the temperature variations observed when droplets impact a cylindrical micropillar array at 200 °C, with a non-embedded fiber layer structure placed atop. The findings indicate that the placement of the fiber membrane on the surface of the micropillar structure leads to extended spreading and evaporation times for the liquid film, attributed to the hydrophilic properties of the fiber membrane. This setup increases the thermal resistance at the micropillar surface, as depicted in the right figure of Figure 11, showing a prolonged temperature decrease due to the presence of the fiber film.

Quantitatively, the temperature profile for the arrangement without the membrane, as shown in the left figure of Figure 11, reveals a decrease in the internal temperature of the ceramic sheet from 195.5 °C to 182 °C within 0.4 s. Conversely, when the fiber membrane is applied atop the micropillar structure, the internal temperature of the ceramic sheet decreases more gradually, from 192.5 °C to 187.5 °C, over 1.2 s, demonstrating the significant impact of the membrane’s hydrophilicity on heat transfer dynamics. This suggests that the direct impact of the droplet on the fiber membrane, followed by heat transfer to the micropillar and subsequently to the ceramic sheet, extends the thermal interaction time to thrice that observed in the absence of a membrane. This fiber membrane configuration effectively impedes further heat transfer, as indicated by the minor temperature fluctuations on the wall surface over the observed time span. Utilizing the heat flow density equation, the heat flux density under these conditions is calculated to be approximately 15,372 W/m^2^.

**Figure 12:**
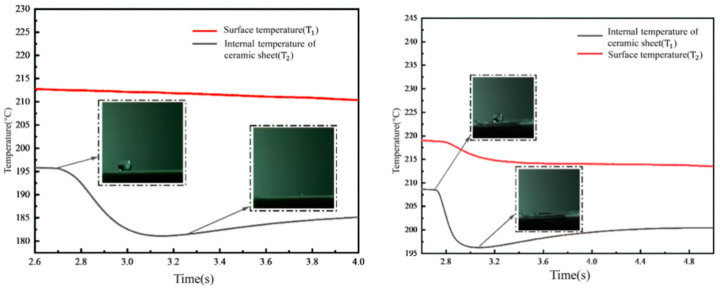
Temperature variation of droplet impacting cylindrical micropillar array with fibrous layer superimposed structure at 200 °C (membrane mosaic).

Figure 12 captures the temperature variations following droplet impacts on a cylindrical micropillar array, with a fiber layer integrated into the structure, all conducted at a temperature of 200 °C (with the membrane integrated in a mosaic pattern). The experiments demonstrate that when a 2 mm diameter droplet impacts the structure at a velocity of 2.23 m/s, the vapor film formation is inhibited by the combined presence of the fiber membrane and the micropillar structure. This arrangement forces the vapor to escape through the flow channels of the cylindrical array, resulting in a distinct step-like profile in both the internal temperature of the ceramic sheet and the wall surface temperature change.

Quantitatively, the embedding of the fiber membrane within the microcolumn structure leads to a noticeable reduction in the internal temperature of the ceramic sheet, from 208 °C to 196 °C within 0.25 s. This is in stark contrast to the slower temperature change observed in the ceramic sheet of the cylindrical array without a membrane, which occurs over 0.4 s. The embedding of the fiber membrane into the cylindrical structure makes the temperature change within the ceramic sheet more pronounced. Additionally, the wall temperature profile reveals that due to the presence of the fiber membrane, a portion of the vapor escapes through the flow channels of the cylindrical array, transferring heat from the micropillar array structure to the base of the ceramic sheet. Since the Leidenfrost phenomenon is not observed, a step change is also evident in the wall temperature curve. Utilizing the formula for calculating heat flow density, the heat flux density under these conditions is estimated to be approximately 10,136 W/m^2^.

**Figure 13:**
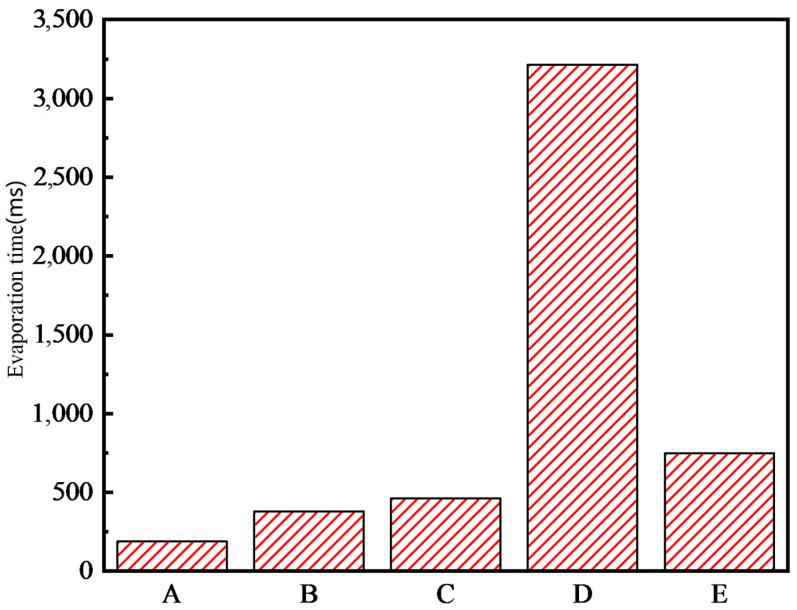
Droplet evaporation times for rectangular microcolumn arrays and cylindrical arrays with different fiber layer arrangements.

Figure 13 displays the evaporation times of droplets interacting with rectangular micropillar arrays and cylindrical arrays, each with varying fiber layer arrangements. In this figure, ‘A’ corresponds to the rectangular micropillar array 2 structure, ‘B’ corresponds to the same array with an added fiber layer, ‘C’ represents the cylindrical array structure, ‘D’ refers to the cylindrical array with a fiber layer positioned atop the micropillars, and ‘E’ depicts the cylindrical array with the fiber layer embedded within the micropillars.

A comparative analysis of the evaporation times of droplets impacting the rectangular micropillar array 2, both with and without a fiber layer, reveals that the fiber membrane prolongs the droplet’s presence on the surface. This is attributed to the enhanced adhesion of the droplet, leading to an extended evaporation duration. Similarly, for the cylindrical array, the addition of a fiber membrane on the surface also results in longer evaporation times. The variance in evaporation durations between configurations ‘D’ and ‘E’ can be traced back to the differing placements of the fiber layer. When the fiber layer is situated on the surface, its slight separation from the micropillar, coupled with its superior hydrophilicity, facilitates the droplet’s spread into a liquid film on the fiber layer. This, in turn, extends the evaporation time as the droplet persists until complete dryness on the membrane. In summary, the introduction of the fiber layer modifies the surface wettability of the microcolumn structure and, as a result, slightly prolongs the total evaporation time for the droplet compared to the surface of the microcolumn structure alone.

## 4. Conclusions

In this paper, we conducted an experimental study to explore the impact of a single droplet impacting the wall of a high-temperature microcolumn array composite structure incorporating a fiber membrane. By altering various experimental parameters, such as the shape of the micropillar array and the placement of the fiber membrane, we observed the motion behaviors of the droplet, including collision, spreading, and splashing, and calculated parameters related to the heat transfer characteristics between the droplet and the wall. We quantitatively analyzed the effects of the composite structure made up of rectangular and cylindrical micropillar arrays with fiber membranes positioned differently on the droplet spreading coefficient, evaporation time, and heat flow density. The main conclusions are as follows:

- Overall, the magnitude of the droplet spreading coefficient is mainly related to the arrangement of the fiber membrane. Since the fiber membrane will change the surface morphology of the microcolumn array, which in turn will change the droplet adhesion, the droplet spreading coefficient will also be changed. When the fiber membrane is arranged at the top of the microcolumn structure, the spreading coefficient is increased by 43% to 46% compared to no fiber membrane. When the fiber membrane is placed on the inside of the microcolumn structure, the spreading coefficient increases by 20% to 33% compared to no fiber membrane.
- The wall surface temperature and the internal temperature of the ceramic sheet are also mainly related to the arrangement of the fiber membrane. The water absorption of the fiber membrane changes the wettability of the surface of the microcolumn structure. When the fiber membrane is arranged in the interior of the micropillar structure, the Leidenfrost effect cannot be formed; the droplets are absorbed by the fiber membrane, and the internal temperature of the ceramic sheet changes drastically by 13.5 °C in 0.4 s. When the fiber membrane is arranged at the top of the microcolumn structure, the droplets are prevented from entering the ceramic sheet, evaporation is slow, and the temperature changes relatively slowly by 5 °C in 1.2 s. The evaporation time of the droplets is strongly influenced by the way the fiber layer is arranged, and the total evaporation time of the fiber layer placed on the top is about 4.3 times that of the fiber layer embedded inside the micropillar structure.
- Increasing the hygroscopicity of micron-structured surfaces via different arrangements of fiber membranes can effectively improve the droplet spreading coefficient and evaporation time, thus increasing the heat flux density. Therefore, the spray cooling performance can be enhanced by combining fiber membranes with microcolumn arrays.

## Figures and Tables

**Table 1:** Error and uncertainty analysis.

Sources of Error	Error Value (°C)	Relative Uncertainties (%)
Thermocouple error	±0.5	0.75%
Droplet impact distance	—	0.39%
Droplet impact time	—	0.11%
Droplet velocity	—	0.20%
Droplet size	—	0.03%
Heat flow	—	2.34%

**Table 2:** Structural parameters of different microcolumn arrays on the wall.

Microcolumn Array Structures	Height (μm)	Diameter (μm)	Spacing (μm)
Rectangular micropillar array	200	200	450
Cylindrical micropillar array	450	200	400

**Table 3:** Test conditions.

	Micro Columnar Structure	Arrangement	Droplet Diameter (mm)	Impact Velocity (m/s)	Wall Temperature (°C)
1	Rectangular micropillar array	No fiber membrane	2 ± 0.2	2.23	200 ± 10
2	Rectangular micropillar array	Fiber membrane on top	2 ± 0.2	2.23	200 ± 10
3	Rectangular micropillar array	Fiber membrane placed inside	2 ± 0.2	2.23	200 ± 10
4	Cylindrical micropillar array	No fiber membrane	2 ± 0.2	2.23	200 ± 10
5	Cylindrical micropillar array	Fiber membrane on top	2 ± 0.2	2.23	200 ± 10
6	Cylindrical micropillar array	Fiber membrane placed inside	2 ± 0.2	2.23	200 ± 10

**Table 4:** Physical properties of high-temperature ceramics.

Sample Name	Materials	Density (Kg/m^3^)	Specific Heat Capacity KJ/(Kg·K)	Thermal Conductivity (W/m·K)
High-temperature ceramics	Aluminum oxide	3819.40	2.27	1.79

## Data Availability

Data are contained within the article.

## Nomenclature

$W_{e}$	Weber number for liquid droplets.
$R_{e}$	Droplet Reynolds number.
α	Angle of wall inclination.
$\sigma_{y}$	Absolute uncertainty of the *y* variable.
$\frac{\sigma_{y}}{y}$	Relative uncertainty of the *y* variable.
∆l	Distance from the droplet to the wall.
∆t	Time from the droplet to the wall.
$T_{w}$	Wall temperature.
v	Droplet velocity.
$D_{e}$	Droplet equivalent diameter.
$D_{h}$	Average droplet horizontal diameter.
$D_{v}$	Average droplet vertical diameter.
q	Heat flux.
P	Thermal power input.
A	Ideal heat transfer surface.
$D^{*}$	Dimensionless spreading diameter coefficient.
$D_{t}$	The instantaneous spreading diameter.
$D_{0}$	Instantaneous diameter of the droplet upon impact with the wall.
$t^{*}$	Dimensionless time coefficient.
$t_{0}$	Time of droplet expansion to maximum diameter.
λ	Thermal conductivity of high-temperature ceramics.
L	Vertical distance from the ceramic sheet’s bottom to its internal temperature measurement point.
